# Synthetic Peptide Fragments of the Wtx Toxin Reduce Blood Pressure in Rats under General Anesthesia

**DOI:** 10.1134/S1607672923700497

**Published:** 2023-09-12

**Authors:** M. S. Severyukhina, A. M. Ismailova, E. R. Shaykhutdinova, I. A. Dyachenko, N. S. Egorova, A. N. Murashev, V. I. Tsetlin, Yu. N. Utkin

**Affiliations:** 1 Branch of Shemyakin–Ovchinnikov Institute of Bioorganic Chemistry, Russian Academy of Sciences, Pushchino, Russia; 2 Pushchino State Natural-Science Institute, Pushchino, Russia; 3 Shemyakin–Ovchinnikov Institute of Bioorganic Chemistry, Russian Academy of Sciences, Moscow, Russia

**Keywords:** non-conventional toxin, WTX, blood pressure, heart rate

## Abstract

Previously, it was shown that the non-conventional toxin WTX from the venom of the cobra *Naja kaouthia*, when administered intravenously, caused a decrease in blood pressure (BP) and an increase in heart rate (HR) in rats [13]. To identify the site of the toxin molecule responsible for these effects, we studied the influence of synthetic peptide fragments of the WTX on BP and HR in normotensive male Sprague–Dawley rats under general anesthesia induced by Telazol and Xylazine. It was found that peptides corresponding to the WTX central polypeptide loop, stabilized by a disulfide bond, at intravenous injection at concentrations from 0.1 to 1.0 mg/mL caused a dose-dependent decrease in BP, with the HR increasing only in the first 5–10 min after administration. Thus, WTX fragments corresponding to the central polypeptide loop reproduce the decrease in blood pressure caused by the toxin.

## INTRODUCTION

**Fig. 1.**
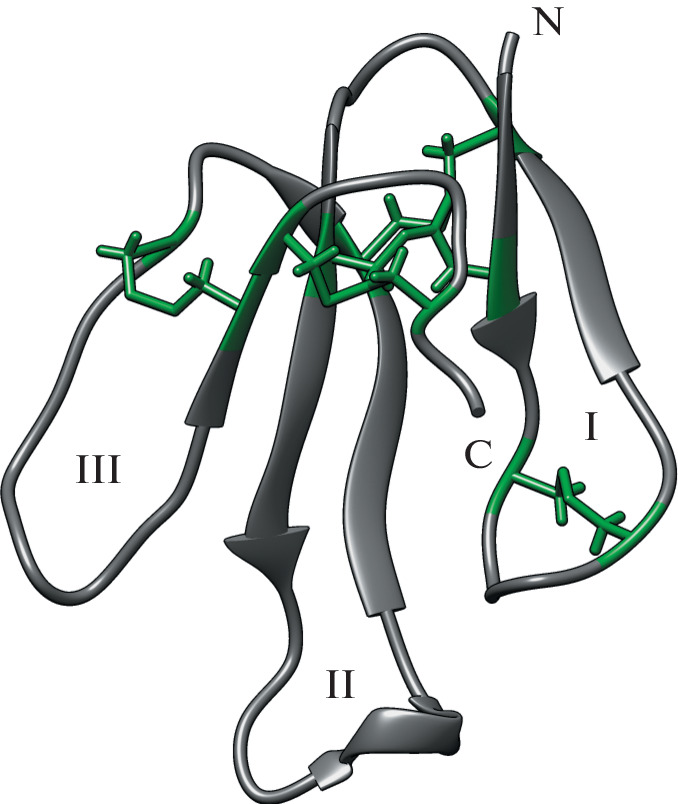
Spatial structure of the toxin WTX (analogue P33A) determined by NMR (PDB code 2MJ0). N and C denote N- and C-terminal residues, respectively. Roman numerals indicate the numbers of polypeptide loops. Disulfide bonds are shown as rods (highlighted in green).

The WTX toxin from the venom of the cobra *Naja kaouthia* belongs to the family of three-finger toxins [1, 2], consists of 65 amino acid residues, and contains five disulfide bonds (Fig. 1) [3]. One of the disulfides is located in the N-terminal loop, in a position characteristic of non-conventional snake toxins [4]. WTX exhibits the properties of both snake α-neurotoxins and muscarinic toxins: it blocks nicotinic acetylcholine receptors (nAChR) of muscle (αβγδ) and α7 type, as well as allosterically interacts with muscarinic acetylcholine receptors [5]. Previously, it was shown that blood pressure (BP) and heart rate (HR) directly depend on the function of nAChR, and their activation or blockade by various agonists or antagonists leads to changes in hemodynamic parameters [6].

The data available to date indicate that the WTX central loop II (Fig. 1) makes the greatest contribution to the interaction with nAChR [7, 8]. This loop interacts with the acetylcholine-binding pocket of nAChR, whereas loop I presumably interacts with the membrane surrounding the receptor [8]. An important role of loop II in the interaction with nAChR was previously shown for snake α-neurotoxins [9, 10]. It was also shown that the WTX loop II plays a major role in interaction with the muscarinic acetylcholine receptor [11], which is consistent with the data for muscarinic toxins [12].

It was previously established that intravenous injection of WTX causes a dose-dependent decrease in BP and an increase in HR, and cholinergic receptors are involved in this process [13]. Taking into account the fact that the WTX polypeptide loop II is involved in the interaction of the toxin with acetylcholine receptors, we decided to test whether the peptide fragments of the WTX amino acid sequence corresponding to this loop would affect BP and HR. It should be noted that a synthetic fragment containing loop II cyclized with disulfide bonds was previously obtained for the cobra *N. naja philippinensis* neurotoxin [14]. The peptide fragment retained the ability to interact with nAChR, which is characteristic of the original toxin, although much less efficiently. Two peptide fragments of the WTX toxin (WTXf1 and WTXf2), the N- and C-terminal regions of which are connected by disulfide bonds, were synthesized by the peptide synthesis, and the effect of these peptides on BP and HR of rats under general anesthesia was studied.

## MATERIALS AND METHODS

Peptides WTXf1 (17 aa) and WTXf2 (21 aa) were obtained by solid-phase synthesis using the procedure that was used previously for the synthesis of a fragment of the central loop of human three-loop protein; the spatial structure of the fragment was fixed by introducing disulfide [15]. The peptide purity was confirmed by analytical HPLC, and molecular masses were determined by mass spectrometry. The molecular masses of WTXf1 and WTXf2 were 2240.2 and 2618.4 Da, respectively, which corresponds to the calculated values within the measurement error.

Male outbred Sprague–Dawley rats of SPF status weighing 250–300 g (Animal Breeding Facility of the Institute of Bioorganic Chemistry of the Russian Academy of Sciences) were used in the experiments. For the study, animals with an initial BP value in the range of 100–140 mmHg., corresponding to the norm, were selected [16]. Animals anesthetized with telazol (4 mg/kg) and xylazine (12 mg/kg, intramuscularly) [17] were implanted with catheters into the common carotid artery and jugular vein through an incision on the ventral side of the neck. The test compounds and the solvent (saline) were administered through an intravenous catheter in a volume of 1 mL/kg. For direct recording of BP and HR, the arterial catheter was connected to a Powerlab ML125 instrument (AD Instrument, Australia).

Animals were divided into seven groups: group 1, control injected with saline (*n* = 6); groups 2–4, injection of WTXf1 at concentrations of 0.1 mg/kg (*n* = 6), 0.3 mg/kg (*n* = 3), and 1 mg/kg (*n* = 1), respectively; groups 5–7, injection of WTXf2 at concentrations of 0.1 mg/kg (*n* = 3), 0.3 mg/kg (*n* = 6), and 1 mg/kg (*n* = 3), respectively. The experiment was performed under general anesthesia (telazol + xylazine). After 15 min of recording the baseline values of BP and HR, the animals were injected through the venous catheter with the drug according to their group affiliation, and the parameters were further recorded for 90 min after the injection of the compounds.

## RESULTS AND DISCUSSION

In previous experiments, rats were injected with WTX at doses of 0.5, 1, and 2 mg/kg [13]. We started the study of the activity of synthetic peptides with a dose of 1 mg/kg. When the WTXf1 peptide was injected to an animal at this dose, the pressure catastrophically dropped to 40 mmHg, which was accompanied by the death of the animal. For this reason, a more detailed study of WTXf1 at a dose of 1 mg/kg was not performed.

**Fig. 2.**
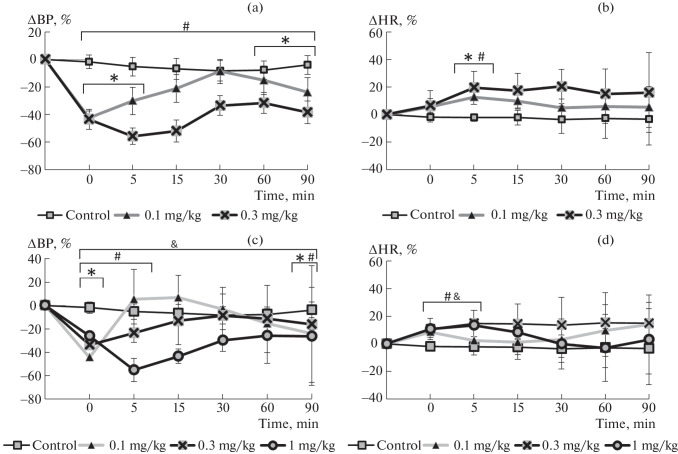
Changes in BP (a, c) and HR (b, d) after intravenous administration of peptides WTXf1 (a, b) and WTXf2 (c, d) to rats. * *p* ≤ 0.05 according to the Mann–Whitney *U* test for the WTXf1 (0.1 mg/kg) and WTXf2 (0.1 mg/kg) groups compared with the control group; ^#^ *p* ≤ 0.05 according to the Mann–Whitney *U* test for the WTXf1 (0.3 mg/kg) and WTXf2 (0.3 mg/kg) groups compared with the control group. ^&^ *p* ≤ 0.05 according to the Mann–Whitney *U* test of the WTXf2 group (1 mg/kg) compared with the control group.

When WTXf1 was injected at a dose of 0.3 mg/kg, a sharp drop in blood pressure by more than 50% was observed in the first 5 min, after which it gradually increased, but remained 30% lower than the baseline values. Approximately 30 min after the injection, another decline in BP began, and by the end of recording the values were also 45% lower than the baseline value (Fig. 2a). It should be noted that two animals from this group died immediately after the experiment, and further study of WTXf1 at this dose was stopped.

Injection of WTXf1 at a dose of 0.1 mg/kg caused a sharp decrease in BP by 40% below the initial value; however, by the 25th minute it returned almost to the baseline level. After the 25th minute of recording, BP significantly decreased and remained stably reduced by 25% below the baseline level until the end of recording (Fig. 2a).

During the entire recording period, no significant changes in BP in the control animals were observed.

The injection of WTXf2 at a dose of 1 mg/kg led to a significant decrease in BP by 45% from the baseline values at the 5th minute. Until the 40th minute, the BP tended to recover, but remained significantly reduced by 27% from the baseline level until the end of recording (Fig. 2c). The injection of WTXf2 at a dose of 0.3 mg/kg caused a drop in BP by 31% in the first minute of recording, followed by its recovery to 87% of the baseline value at the 20th minute. Then, BP remained significantly reduced until the end of recording (80–83% of the baseline values by the 90th minute) (Fig. 2c). The injection of WTXf2 at a dose of 0.1 mg/kg led to a 45% decrease in BP in the first minute, followed by recovery to the baseline value within 3–4 min. Then, a short-term increase in BP by 5% was observed, which was not statistically significant and lasted for up to 15 min after the drug injection. Thereafter, BP gradually decreased by 26% from the baseline level at the end of recording (Fig. 2c).

In the control group, the injection of saline caused no statistically significant changes in HR during the entire recording period.

In the groups injected with WTXf1, a statistically significant increase in HR relative to the control group was observed at 5‒10 min of recording at a dose of 0.1 mg/kg and at 5 min at a dose of 0.3 mg/kg (Fig. 2b). In the groups injected with WTXf1 at doses of 0.3 and 1 mg/kg, the HR increased up to the 5th  minute of recording (Fig. 2d), whereas in the group injected with WTXf2 at a dose of 0.1 mg/kg, no statistically significant differences relative to the control group were observed.

**Fig. 3.**
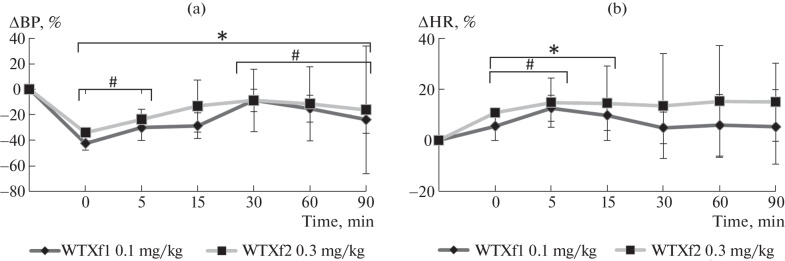
Comparison of the effects of WTXf1 and WTXf2 at doses of 0.1 and 0.3 mg/kg, respectively, on BP (a) and HR (b). * *p* ≤ 0.05 for WTXf1 at 0.1 mg/kg compared with baseline (*t* test for dependent samples), # *p* ≤ 0.05 for WTXf2 at 0.3 mg/kg compared with baseline (*t* test for dependent samples).

The data obtained indicate that doses of 0.1 and 0.3 mg/kg for WTXf1 and WTXf2, respectively, are the most promising for further more detailed studies. We compared the effect of these doses on BP and HR (Fig. 3). In general, the observed effects are similar. However, there are also some differences. For example, for WTXf1 at a dose of 0.1 mg/kg, a statistically significant decrease in BP was observed throughout the experiment, whereas WTXf2 at a dose of 0.3 mg/kg caused a statistically significant decrease in BP from the 1st to the 10th minute and from the 30th minutes to the end of recording (Fig. 3a). A statistically significant increase in HR relative to the baseline values for WTXf1 at a dose of 0.1 mg/kg was observed from the 1st to the 20th minute after injection; for WTXf2, at a dose of 0.3 mg/kg only from the 1st to the 5th minute (Fig. 3b). Thus, the effect of WTXf1 is more pronounced and requires lower doses to achieve similar changes in BP compared to WTXf2. However, WTXf1 at doses of 0.3 and 1 mg/kg under anesthesia stably decreases BP and exhibits toxic properties. A critical decrease in BP may indicate a dose-dependent inhibitory action of WTXf1 on the vasoconstrictor effect of the sympathetic nervous system [18]. Interestingly, the effective dose of WTXf1 (0.1 mg/kg = 0.045 µmol/kg) is significantly lower than that that of the known low-molecular-weight drugs (6.0–15.0 µmol/kg for phentolamine [19, 20] and 20.0–80.0 µmol/kg for captopril [21]).

## CONCLUSIONS

The results of the study of the action of two WTX fragments at different doses on Sprague–Dawley rats under anesthesia revealed a dose-dependent hypotensive effect starting from the first minute of injection, which indicates the ability of each of the fragments to have a direct myotropic effect on the vasculature. It was also found that WTX fragments statistically significantly increased HR in the first 5–10 min after injection, with subsequent rapid return to the baseline values. Thus, WTX fragments corresponding to the amino acid sequence of the central loop of the toxin exhibit the hypotensive effect that was observed previously for the whole toxin [13]. Since the studied peptides account for less than 1/3 of the WTX amino acid sequence, they are more promising as a basis for creating drugs than the toxin itself. This especially applies to the shorter WTXf1 peptide, which, in addition, exhibits a greater activity.
