# Erratum to: **Synthetic Peptide Fragments of the Wtx Toxin Reduce Blood Pressure in Rats under General Anesthesia**

**DOI:** 10.1134/S1607672923050095

**Published:** 2024-01-24

**Authors:** M. S. Severyukhina, A. M. Ismailova, E. R. Shaykhutdinova, I. A. Dyachenko, N. S. Egorova, A. N. Murashev, V. I. Tsetlin, Yu. N. Utkin

**Affiliations:** 1 Branch of Shemyakin–Ovchinnikov Institute of Bioorganic Chemistry, Russian Academy of Sciences, Pushchino, Russia; 2 Pushchino State Natural-Science Institute, Pushchino, Russia; 3 Shemyakin–Ovchinnikov Institute of Bioorganic Chemistry, Russian Academy of Sciences, Moscow, Russia

The article “Synthetic Peptide Fragments of the Wtx Toxin Reduce Blood Pressure in Rats under General Anesthesia,” written by M. S. Severyukhina, A.M. Ismailova, E.R. Shaykhutdinova, I.A. Dyachenko, N.S. Egorova, A.N. Murashev, V.I. Tsetlin, and Yu.N. Utkin, was originally published Online First in Springer-Link on September 12, 2023 without Open Access. After publication, the authors decided to make the article an Open Access publication. Therefore, the copyright of the article has been changed to © The Author(s) 2023 and the article is forthwith distributed under the terms of a Creative Commons Attribution 4.0 International License (http://creativecommons.org/licenses/by/4.0/, CC BY), which permits use, duplication, adaptation, distribution and reproduction of a work in any medium or format, as long as you cite the original author(s) and publication source, provide a link to the Creative Commons license, and indicate if changes were made.

